# Associations of depressive symptoms and social dysfunction with happiness in adults with cardiovascular disease: a cross-sectional study

**DOI:** 10.1186/s40359-025-03044-w

**Published:** 2025-08-29

**Authors:** Towhid Babazadeh, Sakhavat Abolhasani, Khalil Maleki Chollou, Sara Pourrazavi

**Affiliations:** 1 Department of Public Health, Sarab Faculty of Medical Sciences, Sarab, Iran; 2 Department of Clinical Biochemestry, Sarab Faculty of Medical Sciences, East Azerbaijan, Sarab, Iran; 3 Department of Nursing, Sarab Faculty of Medical Sciences, Sarab, Iran; 4 Research Center of Psychiatry and Behavioral Sciences, Tabriz University of Medical Sciences, Tabriz, Iran

**Keywords:** Happiness, Psychological well-being, Self-efficacy, Cardiovascular diseases

## Abstract

**Objective:**

Focusing on the well-being of individuals with chronic conditions, such as cardiovascular diseases, is vital for enhancing their overall quality of life. Hence, this study examined the associations between selected psychosocial factors—namely self-efficacy, depressive symptoms, and social dysfunction—and happiness among patients with cardiovascular disease.

**Methods:**

A total of 150 adult cardiovascular patients completed a questionnaire-based survey in 2023. The survey assessed happiness, psychological well-being, general self-efficacy, and demographic variables. Hierarchical linear regression models were used for data analysis, conducted using SPSS version 21.0 software. A significance level of *P* < 0.05 was considered statistically significant.

**Results:**

In the first step, demographic variables significantly predicted 45.9% of the variance of happiness (*p* < 0.05). In the second step, after adjusting control variables and dimensions of psychological well-being and general self-efficacy, the explained variance increased to 70.7% of the variance by adding variables (*p*-value < 0.05). Depressive symptoms (β= -0.316, *p*-value < 0.05) and social dysfunction (β=-0.204, *p*-value < 0.05) showed the strongest negative associations with happiness in cardiovascular patients.

**Conclusions:**

Given the strong negative associations between depressive symptoms, social dysfunction, and happiness, psychosocial interventions focused on reducing depressive symptoms and improving social functioning may be effective in enhancing well-being among individuals with cardiovascular disease. It is recommended to integrate mental health screening and support into routine cardiac care.

**Supplementary Information:**

The online version contains supplementary material available at 10.1186/s40359-025-03044-w.

## Introduction

Cardiovascular Diseases (CVDs) are one of the main and most common causes of death worldwide, which puts much pressure on healthcare systems [1]. The global burden of CVDs is high [2, 3]. The global trends for disability-adjusted life years (DALYs) and years of life lost due to CVDs have increased significantly, and years lived with disability doubled from 17.7 million to 34.4 million over 1990–2019 [2]. According to global burden of CVDs report (1990–2022) in North Africa and Middle East, after ischemic heart disease and all stroke subtypes, hypertensive heart disease had the highest age-standardized DALYs in 2022 [3]. In Iran, it is estimated that CVDs can lead to 46% of all deaths and 20–23% of disease burden [4]. Various changeable and unchangeable factors, such as genetic, psychological, behavioral, and socio-economic, can play a role in the occurrence or control of CVDs [5]. Among changeable factors, negative emotions have been shown to harm the body’s cardiovascular system, while positive emotions can help it function better [6].

Happiness, a subjective state of well-being, is characterized by positive emotions, contentment, and satisfaction with life [7, 8]. Happiness is protective for physical health and mortality reduction [7]. It directly and indirectly affects CVDs. The risk of death is lower in cardiovascular patients who report higher happiness levels in life [7]. In addition, happiness is associated with healthier behaviors such as eating a balanced diet, being physically active, and getting enough sleep, which helps cardiovascular health [6, 7].

On the other hand, psychological management includes patients’ ability to identify and adjust emotional responses, having emotional vitality and optimism can help individuals manage CVDs or prevent heart attacks and strokes [9, 10]. Indeed, happiness has a protective effect on mental well-being, reducing levels of anxiety and depressive symptoms, which are known risk factors for CVD [11]. This positive emotional state can promote well-being and optimism, leading to better stress management, lower blood pressure, and improved heart health [7]. Overall, being happy can positively impact cardiovascular health’s physical and mental aspects.

Happiness has a complex structure influenced by multiple factors such as psychological and socio-economic [7, 11]. Happiness can activate the body’s immune system by affecting cognitive processes such as self-efficacy and positively affect various aspects of health [11]. Self-efficacy, a person’s belief in her/his ability to perform a behavior successfully [12], is a significant predictive factor for psychological well-being and happiness [1, 11]. It plays a crucial role in shaping one’s emotional experiences and happiness [11]. General health is another factor for which growing evidence suggests that it plays a predictive role in happiness, with a bidirectional relationship between the two. Individuals with better health tend to experience higher levels of happiness, while happiness has been shown positively correlated with general health outcomes [13–15]. Happiness and life satisfaction are associated with better health and fewer health problems such as coronary artery disease, stroke, type 2 diabetes, and longer life span [7, 15–18]. On the other hand, increased levels of perceived illness and severe health problems are associated with lower happiness levels [13].

As mentioned, the importance of happiness in maintaining and improving the health of the body’s cardiovascular system is known [4]. However, despite the fact that the Islamic Republic of Iran has one of the highest standardized rates of CVD, it ranks 101st in terms of happiness in the World Happiness Report [19]. This gap highlights the importance of conducting more localized research on the psychosocial determinants of happiness in Iranian cardiac patients.

The present study is grounded in the biopsychosocial model of health, which posits that psychological (e.g., depressive symptoms), social (e.g., social dysfunction), and personal (e.g., self-efficacy) factors interact with biological conditions to shape health outcomes [20]. Based on this framework, we selected these psychosocial constructs as potential predictors of happiness in cardiovascular patients, in order to answer the question: To what extent are self-efficacy, depressive symptoms, and social dysfunction associated with happiness among adults with CVD? Therefore, this study aimed to examine the associations between self-efficacy, selected dimensions of general health (i.e., depressive symptoms and social dysfunction), and happiness in adult cardiovascular patients.

## Methods

### Participants

This cross-sectional study occurred in 2023 at Emam Khomeini Hospital of Sarab, Iran. The participants were 150 cardiovascular patients selected using the census sampling method and who had consented to participate in the research. To be included in the study, participants had to meet certain criteria, including physical and cognitive ability to participate in the survey process, as assessed by the interviewer. Exclusion criteria encompassed mortality, lack of significant medical records, and voluntary withdrawal from the study. In addition, participants were excluded if they were experiencing a severe exacerbation of CVD symptoms at the time of data collection (e.g., hospitalization or acute clinical instability), and were therefore unable to participate in the survey process. Mild or stable symptoms did not lead to exclusion. This criterion was employed to ensure that participants were able to respond accurately and provide reliable data for the purposes of the cross-sectional analysis. Two trained interviewers collected the data for this study. They took part in a standardized training session prior to the study, which helped to familiarize them with the study protocol and process for collecting data. The training was focused on explaining the purpose of the study, the correct administration of questionnaires, and, more importantly, how to address any potential concerns that may arise during the interviews. Throughout their interactions with participants, the interviewers followed a structured interview protocol in order to maintain some level of consistency. To monitor inter-rater reliability, each interviewer reviewed a subset of completed interviews for cross-checking. Any discrepancies were documented and resolved by the interviewers’ review and discussion in order to ensure consistency of participant responses and reduce the effects of personal or perceived bias.

### Instruments

#### Demographic information

Demographic information includes participants’ age, gender, marital status, occupation status, education level, monthly income status (lower than 100 dollars (weak), 100 to 200 dollars (moderate), more than 200 dollars (good)), history of smoking, nurturing a deep friendship, and taking pleasure in attending social gatherings.

##### Happiness

The researchers in the study [21] utilized a Persian translated version of the Oxford Happiness Questionnaire, which had already been validated for Persian-speaking populations. This questionnaire comprised 29 items, and participants were asked to respond on a 6-point Likert-type scale ranging from ‘strongly disagree’ to ‘strongly agree’. The possible range of scores on the scale was 1 to 6, with higher scores indicating greater happiness levels. The internal consistency of the translated questionnaire was assessed using the alpha coefficient, which yielded an estimated value of 0.90, indicating good internal consistency of the scale.

##### Psychological well-being

The assessment of psychological well-being utilized Goldberg’s General Health Questionnaire (GHQ-28) [22], consisting of 28 items divided into four subscales. These subscales focused on physical symptoms, anxiety/insomnia, social dysfunction, and severe symptoms of depression, with each containing seven items. Participants rated the items on a four-point scale (0–3), where higher scores indicated lower mental health. The Persian version of the questionnaire demonstrated a high estimated alpha coefficient of 0.93 [23].

##### General self-efficacy

In order to assess general self-efficacy, the researchers utilized Sherer’s scale, which consisted of 17 items. Each item was measured using a 5-point Likert- type scale, ranging from 1 = strongly disagree to 5 = strongly agree, with a neutral option [3] in the middle. The total score on the scale could range from 17 to 85, where higher scores indicated higher levels of self-efficacy. The General Self-efficacy Questionnaire was validated in Iran by Charkhabi et al. [24], who reported an alpha coefficient of 0.83, indicating good internal consistency.

Descriptive statistics for the total scores of the Oxford Happiness Questionnaire, GHQ-28, and General Self-Efficacy Scale are presented in Supplementary Table 1.

### Ethics statement

The study was approved by the Sarab Faculty of Medical Sciences Ethics Committee (Code number: IR.SARAB.REC.1402.006). Informed consent was obtained from all participants before completing the survey instrument. The study was conducted in accordance with the principles of the Declaration of Helsinki.

### Data analysis

Percentages and frequencies were used to describe categorical variables, while continuous variables were analyzed using either the mean and standard deviation or the median and interquartile range, depending on the distribution of the data. All statistical analyses were conducted using SPSS version 21.

Independent samples t-tests were applied to compare groups on quantitative variables. The relationships between happiness and variables such as physical symptoms, anxiety/insomnia, social dysfunction, depressive symptoms, and general self-efficacy were examined using Pearson’s correlation coefficient, which ranges from − 1 to 1. The Kolmogorov-Smirnov test was employed to assess the normality of the data.

A hierarchical linear regression was conducted to explore predictors of happiness, using a two-step approach. In the first step, demographic variables—including age group, gender, marital status, income, employment status, education level, smoking history, close friendships, and enjoyment of social events—were entered. In the second step, scores for physical symptoms, anxiety/insomnia, social dysfunction, depressive symptoms, and general self-efficacy were added. The adjusted R² change after each step was calculated to determine the proportion of variance in happiness explained by the model. Assumptions of regression—including multicollinearity, normality, and detection of influential data points—were tested and found to be satisfactory. The Variance Inflation Factor (VIF) index was used to assess the value of multicollinearity between independent variables. A significance threshold of α = 0.05 was used throughout.

## Results

Table 1 shows the demographic information of the participants. The mean (± SD) age of the participants was 57.94 (± 13.43), and approximately half of the participants were aged 65 years or older. About 52.7% of participants (*n* = 79) were females and the majority (94%, *n* = 114) were married. Around 44% of the participants (*n* = 66) reported moderate income. Additionally, Table 1 shows that income (*p*-value = 0.001), education (*p*-value = 0.001), nurturing a deep friendship (*p*-value = 0.001), and taking pleasure in attending social gatherings (*p*-value = 0.001) have statistically significant associations with happiness.

**Table 1:** Demographic characteristics of the patients and their association with happiness

Variable		N (%)	Happiness	*p*-value*
			Me ± SD	
**Age groups [year]**	65 years and older	83 (55.3)	109.16 ± 22.68	0.159
	< 65 years old	67 (44.7)	103.89 ± 22.71	
**Gender**	Male	71 (47.3)	108.23 ± 21.24	0.469
	Female	79 (52.7)	105.53 ± 24.12	
**Marriage**	Single	9 (6.0)	114.55 ± 32.81	0.478
	Married	141 (94.0)	106.31 ± 22.05	
**Income**	Weak	63 (42.0)	98.04 ± 22.84	0.001*
	Moderate	66 (44.0)	109.40 ± 19.04	
	Good	21 (14.0)	124.95 ± 21.39	
**Job**	Office Clerk	56 (37.3)	107.35 ± 21.81	0.717
	self-employment	94 (62.7)	105.91 ± 24.47	
**Education**	Under diploma	97 (64.7)	102.04 ± 22.46	0.001*
	Diploma and higher	53 (35.3)	115.54 ± 20.82	
**History of smoking**	Yes	34 (22.7)	104.41 ± 22.31	0.486
	No	116 (77.3)	107.51 ± 22.95	
**Nurturing a deep friendship**	Yes	98 (65.3)	111.72 ± 22.76	0.001*
	No	52 (34.7)	97.55 ± 19.89	
**Taking pleasure in attending social gatherings**	Yes	91 (60.7)	115.17 ± 19.20	0.001*
	No	59 (29.3)	93.91 ± 21.93	

*P-value < 0.05

The bivariate associations for physical symptoms, anxiety/insomnia, social dysfunction, depressive symptoms, and general self-efficacy with happiness are shown in Table 2. Using the Pearson correlation coefficient test, we discovered that happiness statistically had significant relationships with all variables (*p*-value < 0.05).

**Table 2:** Bivariate correlation matrix of the relationship between general health, self-efficacy, and happiness

Variables	1	2	3	4	5	6
**1 = Physical symptoms**	1					
**2 = Anxiety/insomnia**	0.637*	1				
**3 = Social dysfunction**	0.485*	0.567*	1			
**4 = Depressive symptoms**	0.448*	0.622*	0.556*	1		
**5 = General self-efficacy**	-0.507*	-0.544*	-0.740*	-0.457*	1	
**6 = Happiness**	-0.562*	-0.658*	-0.687*	-0.685*	0.652*	1

*Correlation is significant at *P*-value < 0.05 [two-tailed]

To estimate levels of happiness, hierarchical multiple linear regression analysis was conducted. In the first step, demographic factors emerged as significant predictors of happiness (*p* > 0.05, R² = 0.459), as presented in Table 3. These demographic variables accounted for approximately 45.9% of the variance in happiness (F = 9.690, *p* < 0.001). Within this step, income (β = -0.163), educational level (β = 0.157), and enjoyment of attending social events (β = 0.216) were identified as significant predictors.

**Table 3:** Hierarchical linear regression for happiness through demographic characteristics, general health, self-efficacy

Variables	ß	R2 change	F change	SE	*P*-value *	Collinearity Statistics	
						Tolerance	Tolerance
**Step 1**							
Age groups	-0.053			2.943		0.83	1.87
Gender	0.017			3.374		0.53	1.87
Marriage	0.067			6.315		0.91	1.09
Income	-0.163*	0.459	9.690	2.453	< 0.001	0.77	1.28
Job	0.123			1.582		0.64	1.55
Education	0.157*			3.421		0.79	1.26
History of smoking				3.983		0.75	1.32
Nurturing a deep friendship				3.421		0.76	1.31
Taking pleasure in attending social gatherings	0.216*			3.419		0.78	1.27
**Step 2**							
Age groups	0.011			2.260		0.77	1.28
Gender	0.050			2.611		0.50	1.98
Marriage	0.075			4.628		0.89	1.11
Income	0.059			1.937		0.66	1.50
Job	0.017	0.247	22.255	1.230	< 0.001	0.61	1.63
Education	0.061			2.773		0.72	1.38
History of smoking	0.003			3.041		0.73	1.35
Nurturing a deep friendship	0.042			2.647		0.66	1.50
Taking pleasure in attending social gathering	0.024			2.807		0.63	1.56
Physical symptoms	-0.111			0.379		0.50	1.99
Anxiety/insomnia	-0.056			0.551		0.38	2.55
Social dysfunction	-0.204*			0.460		0.38	2.57
Depressive symptom	-0.316*			0.410		0.46	2.17
General self-efficacy	0.145			0.152		0.43	2.13
Total R2	-	0.707	-	-	-	-	-
Adjusted R2	-	0.669	-	-	-	-	-

*P-value < 0.05

In the subsequent step, additional variables—namely, physical symptoms, anxiety/insomnia, social dysfunction, depressive symptoms, and general self-efficacy—were included in the model. These factors contributed an extra 24.7% to the explained variance (F = 22.255, *p* < 0.001). Altogether, the full model incorporating both demographic and psychological variables accounted for 70.7% of the total variance in happiness (see Table 3).

## Discussion

Happiness is a complex concept due to different perceptions of people and different meanings attributed to it, and various factors play a role in it. Although extensive research has been done on it, there is still no universal definition of happiness for all humans. For example, for patients, health is considered an important element for happiness, and they stated that health is more important for happiness than monetary enrichment. In addition, valuing happiness helps them to live with their illnesses and to accept and adapt to the contradictions in their lives [25]. Among cardiovascular patients promoting happiness as one of the psychological management strategies has an important role in the cardiac rehabilitation of patients [9, 26]. Therefore, this study examined the relationship between self-efficacy, general health dimensions, and happiness. However, it is worth noting that happiness is a multidimensional construct. In addition to the factors examined in this study, other domains such as perceived social support, spirituality, purpose in life, and cultural values ​​can significantly shape individuals’ sense of happiness and well-being [6, 13]. Although the present study focused on selected psychosocial variables, this focus does not negate the influence of other important factors, and future studies should consider a wider range of psychological and cultural variables to achieve a more comprehensive understanding of happiness in patients with CVDs.

The study’s findings indicated that depressive symptoms and social dysfunction were the strongest negative predictors of happiness, after controlling for demographic variables. In the subscales of GHQ, depressive symptoms showed the greatest contribution to the overall score. Depressive symptoms have been associated with an increased risk of coronary heart disease, and many cardiovascular patients experience mental disorders such as anxiety and depressive symptoms [27]. Depressive symptoms are a common and debilitating symptom in chronic diseases [27] that can decrease happiness in patients [28]. Depressive people usually do not enjoy doing happy and entertaining activities or do not have the interest and motivation to participate in those activities [27]. On the other hand, cardiovascular patients have some physical disabilities and symptoms such as fatigue that limit their daily life activities, leisure time, and interaction with family, friends, and colleagues [27, 29]. Reducing personal communication and interactions can reduce happiness in these people [30]. However, studies have shown that vitality, happiness, and mental well-being are related to cardiovascular health [31].

Given that anhedonia is one of the core diagnostic criteria for depressive disorders [32], it’s not surprising that people who experience more depressive symptoms also tend to report feeling less happy. This overlap raises an important question of whether decreased happiness can be considered, in part, as a symptom of depression. Although our findings suggest a strong negative association between depressive symptoms and happiness, this relationship may be somewhat tautological. However, symptoms of depression can include a wider range of emotional and cognitive problems that go beyond a reduction in positive affect, including hopelessness, fatigue, and impaired functioning. Therefore, we believe that including depressive symptoms in the model is still theoretically meaningful, while accepting the conceptual overlap as a limitation of interpretation.

Social dysfunction, as another subscale of general health, which has a negative relationship with happiness, is also common in cardiovascular patients [27]. Social functioning is essential in determining whether cardiovascular patients can return to social life [33]. Social functioning is defined as a person’s ability to play different social roles, interact with family, friends, and colleagues and participate in social activities [27]. Literature has shown that the lack of ability to engage in social roles and participate in social activities can negatively affect the mental well-being and health status of cardiovascular patients [27, 34]. Social functioning occurs based on a person’s physiological and psychological health status. Cardiovascular patients usually experience reduced physical ability. As a result, they are at high risk of social dysfunction [27]. Social dysfunction often related to a gradual withdrawal from relationships and social life, which in turn leads to further aggravation of psychological symptoms [33]. Communication and social interactions can increase happiness [35]. Therefore, factors related to social interactions, loneliness and isolation, lack of job activities, and social dysfunction play an important role in reducing people’s happiness [28].

It is well known that cardiovascular patients suffer from physiological, psychological, and social problems [27]. Social relationships are an important part of everyday life, significantly related to an individual’s subjective well-being [36]. Literature has shown the positive effect of social communication on CVDs, and researchers have investigated the effects of human interactions on heart health. They reported that social connections with friends, family, neighbors, or colleagues increased the chance of survival by 50% [30]. On the other hand, Chang has found that participation in voluntary and involuntary institutions, community participation, and trust increase subjective happiness [37]. In other words, improving social relations and interactions between people is important in defining happiness. Happiness is the result of interaction between people [30]. Therefore, it seems that enhancing social interactions could be a more effective strategy for promoting happiness in CVD patients than focusing exclusively on improving objective life conditions. However, given the cross-sectional nature of the data, longitudinal studies are necessary to further investigate this relationship. Although we reported biological characteristics such as NYHA class and ejection fraction (EF%) of the participants, these variables were not included in the statistical analysis. This omission, as noted in the limitations, may affect the generalizability of our findings to broader CVD populations with different clinical profiles. Future studies could further explore how these biological variables interact with the studied outcomes.

The results of the study showed that the level of happiness increases with education. Studies have shown that happiness has a positive and significant effect on the educational level, and it is considered one of the key goals of education [38, 39]. A higher education gives people more control over their work and life, as a result, they achieve their expectations and goals more effectively, deal with problems more actively and optimistically, and achieves self-improvement and independence in work, and undoubtedly be happier [38]. In addition, high income was also identified as one of the predictors of happiness. High income can expand the range of people’s choices, allowing them to meet their individual preferences and needs better and thus experience a higher level of happiness [38].

According to our findings, targeted interventions that address depressive symptoms and social dysfunction in cardiovascular patients appear to be essential. In this context, the literature has indicated that psychological interventions such as cognitive-behavioral therapy and mindfulness-based stress reduction have been effective in reducing depressive symptoms and improving mental well-being in cardiac populations [40, 41]. In addition, structured social support programs, such as peer support groups, community-based social participation activities, can improve social functioning and overall happiness [42]. Educational sessions aimed at improving self-efficacy and emotion regulation have also been effective in managing the disease and improving their happiness [1, 43]. Incorporating such evidence-based approaches into routine cardiovascular care could play an important role in increasing patients’ happiness and psychological outcomes.

## Limitations

The limitations of the study must be acknowledged. First, the investigation was cross-sectional and relied on the recollection of past events; thus, no causal inferences among happiness and psychosocial variables could be made. Second, while we assumed that participants provided answers honestly; the possibility of socially desirable responses cannot be entirely ruled out. To mitigate this, interviewers were trained and respondents were assured of confidentiality at the start of the survey. Third, the relatively small sample size was not sufficiently powerful to investigate true relationships; studies with larger samples are suggested to be conducted in the future. Fourth, although the study used a biopsychosocial model, it primarily focused on psychological and social factors. Biological variables (such as clinical markers, CVD subtypes, or physiological indices) were not included in this study due to the cross-sectional and self-report design of the study, as well as data limitations. The biological components, along with psychosocial factors, should considered merit in future studies aimed at understanding the determinants of happiness in patients with CVD. Fifth, it is important to note that the variable of happiness is multidimensional and is influenced by numerous factors including: psychological, biological, cultural, spiritual, and environmental. In this research, focus was on selected psychosocial factors particularly self-efficacy, depressive symptoms, and social functioning. Such limited focus could be overly reductionist in focusing only on aspects of happiness, and not capture the full range of constituents that enhance well-being. Other contextual and cultural factors beyond psychosocial components would enrich the happiness framework and should be included in future studies. Sixth, although depressive symptoms, social dysfunction, and happiness were assessed as distinct constructs, it is likely they have conceptually and psychologically overlap. Their high intercorrelations pose concerns regarding their independence. This could complicate interpreting the unique variance ascribed to each factor and may strengthen assumed interrelationships. More precise measurement approaches or latent variable modeling could be better suited to examine these questions to eliminate being overly simplified, or minimize overly assuming such constructs. Seventh, for the purposes of this study, respondents were placed into broad groups based on their self-reported occupational titles. While this method simplified certain statistical analyses, it likely missed some important within-group variation. More precise and uniform occupational classifications are necessary for enhanced interpretation in subsequent studies. Eighth, the study lacked detailed clinical information on CVD diagnoses, duration of disease, or severity (e.g., ejection fraction, NYHA class). The absence of this information prevents identifying whether the psychosocial factors we identified function similarly across all levels of disease severity. Future studies should include clinical parameters to help better delineate how physical health status may interact with psychological health status.

## Conclusion

The current study investigated the associations of self-efficacy and the general health dimensions with happiness in cardiovascular patients. The study demonstrated that depressive symptoms and social dysfunction as two dimensions of general health were the strongest predictors of happiness in cardiovascular patients. Considering that cardiovascular patients experience problems that reduce their involvement in social interactions and enjoyment of pleasurable activities, they are at increased risk for depressive symptoms and social dysfunction. Measures such as establishing support group meetings for patients and increasing their interaction and sympathy through sharing their experiences can increase the level of happiness in these people.

## Electronic supplementary material

Below is the link to the electronic supplementary material.

Supplementary Material 1

## Data Availability

The data that support the findings of this study are available from the corresponding author, SP, upon reasonable request.
