# Supplementary material for: Associations of depressive symptoms and social dysfunction with happiness in adults with cardiovascular disease: a cross-sectional study
Source: BMC Psychol. 2025 Aug 29;13:980. doi: 10.1186/s40359-025-03044-w (PMC12395655; doi:10.1186/s40359-025-03044-w)
Supplement: Supplementary file 1 — Supplementary Material 1 [file 40359_2025_3044_MOESM1_ESM.doc]

| **Questionnaire** | **Number of Items** | **Mean (SD)** | **Min** | **Max** | **Cronbach’s α** |
| --- | --- | --- | --- | --- | --- |
| Oxford Happiness Questionnaire | 29 | 4.12 (0.76) | 2.1 | 5.9 | 0.90 |
| GHQ-28 Total Score | 28 | 21.85 (8.44) | 4 | 56 | 0.93 |
| General Self-Efficacy | 17 | 61.35 (11.22) | 27 | 83 | 0.83 |

**Supplementary Table 1. Descriptive Statistics for Total Scores of the Oxford Happiness Questionnaire, GHQ-28, and General Self-Efficacy Scale among Cardiovascular Patients**
